# Rate of motor progression in Parkinson’s disease: a systematic review and meta-analysis

**DOI:** 10.3389/fneur.2024.1452741

**Published:** 2024-09-26

**Authors:** Ayla Pauwels, Albert L. G. Phan, Catherine Ding, Thanh G. Phan, Peter A. Kempster

**Affiliations:** ^1^Department of Neurology, Monash Health, Melbourne, VIC, Australia; ^2^NEUR Research Group, Center for Neurosciences, Vrije Universiteit Brussel, Brussels, Belgium; ^3^Faculty of Medicine, Dentistry and Health Sciences, The University of Melbourne, Melbourne, VIC, Australia; ^4^Department of Medicine, School of Clinical Sciences at Monash Health, Monash University, Melbourne, VIC, Australia

**Keywords:** Parkinson’s disease, levodopa, progression, natural history, meta-analysis

## Abstract

**Background:**

The search for neuroprotective treatments for Parkinson’s disease (PD) still relies largely on motor disability scales. A limitation of these tools is the strong influence of symptomatic dopaminergic treatment effects. Drawing on a wealth of published information, we conducted a systematic review and meta-analysis of motor progression in PD and its relationships with dopaminergic therapy.

**Methods:**

We searched Medline, Embase, and Central to identify 84 publications with adequate serial motor scores to calculate progression, expressed as an increase in the percentage of maximum disability.

**Results:**

A random-effects model showed motor progression at 2.0% p.a. (95% CI 1.7–2.4%). There were no significant differences by baseline age, sample size, or observation period. However, untreated patients, in 8 publications, progressed at 4.5% p.a. compared to 1.6% p.a. in 76 studies containing individuals on dopaminergic drugs (*p* = 0.0004, *q* = 0.003). This was supported by research on phenoconversion in prodromal PD, where motor progression exceeded 5% p.a. in the 2 years before diagnosis. Starting levodopa improved pre-treatment disability by 40.3 ± 15.2%. Practically defined *off* state measurements increase faster than *on* scores by a modest degree (*p* = 0.05).

**Conclusion:**

This survey suggests that accurate long-term measurements of motor progression to assess disease-modifying therapies can be conducted despite the sequential commencement of dopaminergic drugs and sample attrition over time. While study designs involving prodromal or untreated PD avoid confounding effects of symptomatic treatment, different assumptions about motor progression may be needed. A defined *off* state with the levodopa test dose method maximizes information about the medication cycle once dopaminergic therapy has begun.

## 1 Introduction

While there are effective symptomatic drug treatments for Parkinson’s disease (PD), there is no proven way to alter the slow progression of its underlying pathology. A 2022 survey identified more than 150 clinical trials of neuroprotective agents (1). None have convincingly shown a disease-modifying effect. Objective motor assessment has been the mainstay of clinical trial methodology for PD. It is used to evaluate both symptomatic treatments—pharmacological or non-pharmacological—as well as potential disease-modifying drugs.

Debate about the shortcomings of disease-modifying research in PD has focused on two main areas—drug development, particularly therapeutic target and experimental agent choices, and clinical trial design (2). Trial design issues include population selection (manifest early PD as opposed to possible latent PD, identified by non-motor clinical features or genetic risk) and outcome measurements (3). Measuring motor disability means making allowance for symptomatic effects, both for experimental agents that might have symptomatic and protective benefits and for the ways in which standard dopaminergic treatments influence motor scoring. Inconclusive results of large trials such as DATATOP (4) and ADAGIO (5) may be traced to assumptions about symptomatic treatment effects. The tradition of reliance on objective motor scales has been questioned. Possible alternatives are composite endpoints by weighting scores from multiple scales, the use of wearable sensors to collect electronic movement data (6), and the identification of dependable clinical milestones of disease progression (7). Laboratory biomarkers as symptom-unrelated outcome measures would be ideal (8). None as yet are sufficiently sensitive to disease progression.

The Braak model of progression of alpha-synuclein pathology explains why olfactory or sleep-related non-motor symptoms can be premonitory PD features, and why cognitive and neuropsychiatric non-motor symptoms attend its advanced stages (9). However, at its core, PD is a motor disorder, and quantitative histological studies show why this is true. Lewy pathology, containing aggregated alpha-synuclein protein, denotes populations of neurons that are selectively vulnerable in PD. These are anatomically and neurochemically diverse, though there are certain morphological similarities. The pars compacta region of the substantia nigra is the most consistently, most severely affected region. Approximately 50% of these neurons have been lost at the time of first motor symptoms, rising to 90% by the end of the disease course (a small subpopulation always survives) (10). Many other regions are affected and may contribute to non-motor symptoms, but cell loss in relation to Lewy pathology is generally less (11). The UPDRS motor scale shows a linear correlation with neuronal density in the substantia nigra (12). A sufficiently sensitive objective clinical motor scale should still be the best way to prove that an investigational agent modifies the pathological progression of PD.

The influence of symptomatic treatments on the measurement of motor disability needs to be addressed in clinical trial design. One approach has been to try to exploit the pre-treatment phase, allowing pure active and placebo comparative arms. Participants are temporarily denied the benefit of dopaminergic drugs, restricting such trials to short time scales. An alternative is long-term assessments guided by a good understanding of the effects of symptomatic treatments on the measurement scale.

The purpose of this article is to examine rates of progression in the various phases of PD and in relation to the commencement and continuation of dopaminergic therapy. There is a wealth of published data that can inform this inquiry. In a systematic review and meta-analysis of serial motor scoring in PD, we will focus on four main areas:

1. Rate of motor progression, both before and during chronic pharmacological treatment;
2. Influence of timing of dopaminergic medication doses on measurements of motor progression, particularly the use of defined *off* and *on* states;
3. Short- and medium-term effects of commencement of dopaminergic treatment on motor disability scores;
4. Appearance and progression of motor disability in the very earliest stage of PD.

The last two points are highly relevant to future trials involving “preclinical” or “prodromal” PD—individuals with strong risk indicators (clinical, genetic, and neuroimaging) for the development of PD yet no sign of motor manifestation. Neuroprotective agents may have their strongest effects before the neurodegeneration of PD is far advanced, prior to detectable motor signs (3). Actual proof of disease modification, though, will probably rely on the measurement of motor disability when symptomatic treatment effects are present.

## 2 Methods

### 2.1 Study design

The study, a systematic review and meta-analysis, was registered with PROSPERO (CRD42023410326). The Preferred Reporting Items for Systematic Reviews and Meta-Analyses (PRISMA) guidelines were followed (13).

### 2.2 Eligibility criteria

Articles were included if they reported on at least 20 individuals diagnosed with or at risk for idiopathic PD who had serial (two or more) measurements of motor function over a minimum interval of 6 months. These motor assessments had to be performed using a recognized objective PD motor disability scale with a range of 20 or more points (excluding less sensitive instruments such as the 5-point Hoehn and the Yahr scale). Separate reporting of motor scores was required. An article was ineligible if there was no English full-text available, or if only an abstract was published.

All study designs yielding longitudinal data, prospective or retrospective, were considered. This included cohort and population-based studies. For clinical trial research, data from control arm patients receiving standard-of-care pharmacological treatment, levodopa monotherapy, or no medication were included. Excluded were data from patients in a clinical trial or comparison who received a trial pharmacological agent, or a non-pharmacological intervention. The purpose of these restrictions was to focus as much as possible on the natural history of PD before and during normal pharmacological therapy. While we accepted that placebo effects would be present in clinical trial results, we excluded studies where this was excessive, as manifested by motor disability scores that were stable or improved during a period of observation (any calculated progression rate ≤ 0%). Studies that subdivided participants to examine certain characteristics, such as genetic factors, were included as long as the whole sample was representative of PD. In cases where multiple studies reported overlapping observations on the same cohort, we selected the study with the longest follow-up and/or largest sample size, which was generally the most recent study.

### 2.3 Data sources

**Figure 1:**
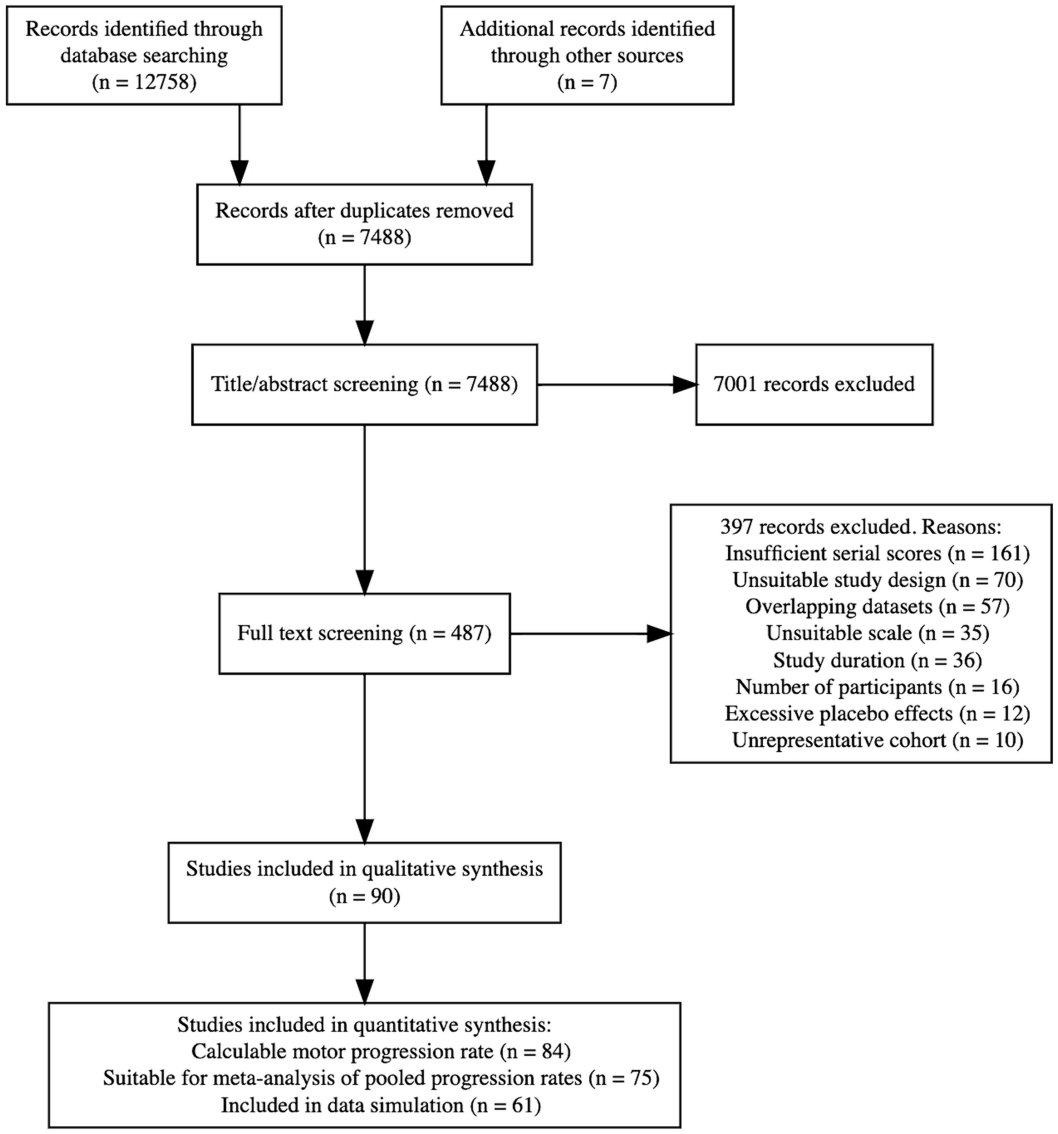
PRISMA flowchart.

On 15 March 2023, we conducted literature searches in Medline (Ovid interface), Embase (Ovid interface), Cochrane Central Register of Clinical Trials, and clinicaltrials.gov. See Appendix 1 for the search strategy and Figure 1 for the PRISMA flowchart.

### 2.4 Article selection and classification

Two authors (AP and PK) independently performed title/abstract and full-text screening to determine eligible papers and categorize key study attributes. In cases of missing information, the nature and cause (if specified) of omissions were registered. Disagreements were discussed with the aim of consensus, and the opinion of the third author (CD) was sought if required.

### 2.5 Data extraction and processing

Data were extracted from tables or graphical plots to obtain the maximum number of time points of motor disability for each paper. In clinical trials with a washout phase at the end of the study period, the washout was excluded. If insufficient data were provided to determine spread—standard deviation (SD) or standard error of the mean—authors were contacted by email to request this information. Where values of two subgroups of PD patients were provided separately (e.g., carriers and non-carriers of a specific gene), the weighted mean was used to calculate the progression rate for the whole sample. One other paper reported on two separate cohorts (14), which both were used.

Where some or all patients received dopaminergic treatment, the relationship of motor scoring with the timing of medication was noted, usually specified as *on* or *off* states. If a study was silent on this point, it was assumed that assessments were performed in prevailing motor states not temporally linked to the medication cycle. The use of practically defined *off* states with levodopa test doses was also recorded. All specified *on* and *off* scoring was used to derive separate progression rates for these motor states. Where a study provided both *on* and *off* scores, an average of *on* and *off* was taken to calculate overall progression. This was done to avoid over-weighting a cohort by double representation, or one type of motor assessment at the expense of the other.

Response to commencement of levodopa therapy was ascertained from studies that met the following conditions: (i) pre-treatment motor disability recorded; (ii) levodopa started at a single time point; (iii) ≥3 motor measurements conducted during the first 2 years of treatment. The initial treatment response was calculated by expressing the amount of motor improvement at the time point of the lowest recorded disability score after starting treatment as a percentage of pre-treatment disability. This nadir of disability was then used as the first point for the calculation of motor progression in those articles.

Three additional classifications of articles were made:

- *Sequential treatment commencement > 25%*: a study in which dopaminergic treatment was not commenced at a single time point but it was estimated that more than 25% of participants had initiated treatment during a study period.
- *Fixed sample size*: a study with the same number of subjects at baseline and final observation point (retrospective cohort analyses).
- *Sample attrition*: a study where the number at baseline exceeded the final observation point because of loss to follow-up (clinical trial and prospective cohort research).

### 2.6 Statistical methods

Statistical analyses were performed with R, version 4.3.1. The raw data were converted to mean ± SD as this was the effect size measure most commonly reported. The median and interquartile range (IQR) were changed to mean and SD using the methods of Luo and Wan (15, 16). SD was obtained from the 95% confidence interval (CI) by dividing it by 3.92 before multiplying it by the square root of the sample size (17). For sample attrition studies that did not provide a patient number for every time point, we assumed a constant attrition rate based on sample size at the first and last time points. If the SD of the rate of progression was not given, it was imputed from the first and last measurements, assuming a conservative correlation coefficient of 0.7 (17, 18). Descriptive statistics are presented as mean ± SD or median ± IQR.

The progression rate in individual articles was determined by linear regression. To standardize the various motor scoring systems, the results are presented as percentage change per annum (p.a.) of the maximum disability score of the relevant scale (19). All studies with a calculable rate of PD motor progression were analyzed according to study attributes using the Wilcoxon rank-sum test with correction for multiple comparisons following the Benjamini-Hochberg principle, with a *p*-value adjusted for false discovery rate (*q*-value) of ≤0.05 considered significant. As an additional check for bias from sample size, study duration, and number of time points, we calculated annual motor progression weighted for each of these factors.

All studies yielding statistics on both the rate of motor progression and its standard error were pooled in a meta-analysis to synthesize evidence and assess for heterogeneity. We employed a DerSimonian and Laird random-effects meta-analysis model, using the meta package in R (20). Heterogeneity between studies was assessed with the I^2^ metric. Study characteristics and bias assessment were explored as potential sources for heterogeneity using multivariate linear models (rma.mv function, metafor package) (21).

Studies with mean and SD statistics on serial time points contributed to a data simulation. We used the truncnorm package (22), with a truncated normal distribution model (range 0 to 100). For studies providing both *on* and *off* scores, the *off* measurements were simulated.

### 2.7 Study quality assessment

Quality was independently assessed by two authors (AP and PK), using the Critical Appraisal and Skills Programme (CASP) quality assessment tool (23). We adapted the cohort study version of the CASP tool and examined all studies for their longitudinal cohort properties, irrespective of the research question or method.

Studies were critically assessed on five points: 1. study population; 2. selection bias; 3. ascertainment of diagnosis; 4. outcomes; 5. results. Ascertainment of diagnosis refers to the use of published PD diagnostic criteria. See Supplementary Table S1 for a detailed overview of our applied scoring system.

## 3 Results

### 3.1 Search results

Figure 1 shows the disposition of 12,765 initially identified articles in a PRISMA flowchart. Following a full-text review, 90 studies remained for qualitative analysis. Of these, 84 studies had a calculable progression rate, 75 had statistics on the spread of the progression rate and were suitable for meta-analysis of pooled results, and 61 allowed raw progression data simulation. All included studies (4, 5, 14, 19, 24–108) are summarized in Supplementary Table S2.

### 3.2 Rate of motor progression of PD

At baseline in studies with a calculable rate of motor progression, the mean age was 63.4 ± 4.1 and the median PD duration was 2.4 ± 3.9 years. The median study duration was 3.0 ± 3.5 years, and the median sample size was 92 ± 159. We estimated that that our article selection encompassed approximately 5.7 × 10^4^ patient-years of PD progression. The following motor scoring systems were used to calculate the progression rate: UPDRS-III (including modifications) (109), 54 studies; MDS-UPDRS-III (110), 20 studies; Webster Rating Scale (including modifications) (111), 6 studies; Columbia University Rating Scale (including modifications) (112), 3 studies; and Scales for Outcomes in Parkinson’s disease (113), 1 study. Eligible studies employed different ways to present their motor progression data. We show progression rates in three formats to maximize the amount of source data and to make the best use of information on statistical spread, where provided.

#### 3.2.1 All progression rates

The average increase of the maximum disability score per year in all 84 studies (including 85 cohorts) with a calculable motor progression rate was 2.2% (median = 1.7 ± 1.6%).

#### 3.2.2 Progression rate by random-effects modeling

**Figure 2:**
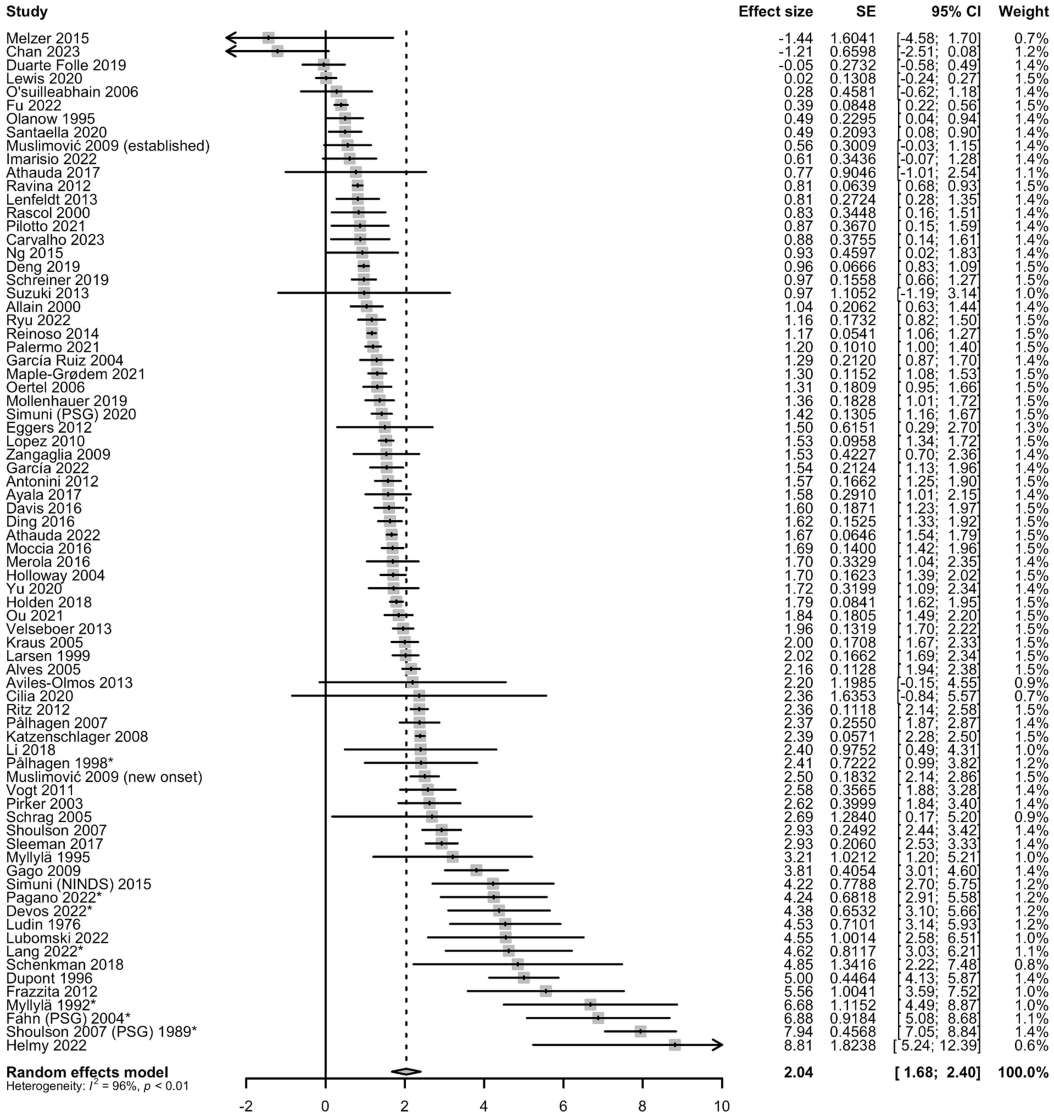
Random forest plot with pooled annual percentage change of maximum disability score. *Articles with untreated patients; SE, standard error.

Enough data were obtained from 75 articles for random-effects modeling. The pooled rate of change was 2.0% (95% CI = 1.7–2.4%) of the maximum disability score per year. This is shown in the forest plot (Figure 2).

#### 3.2.3 Data simulation approach

**Figure 3:**
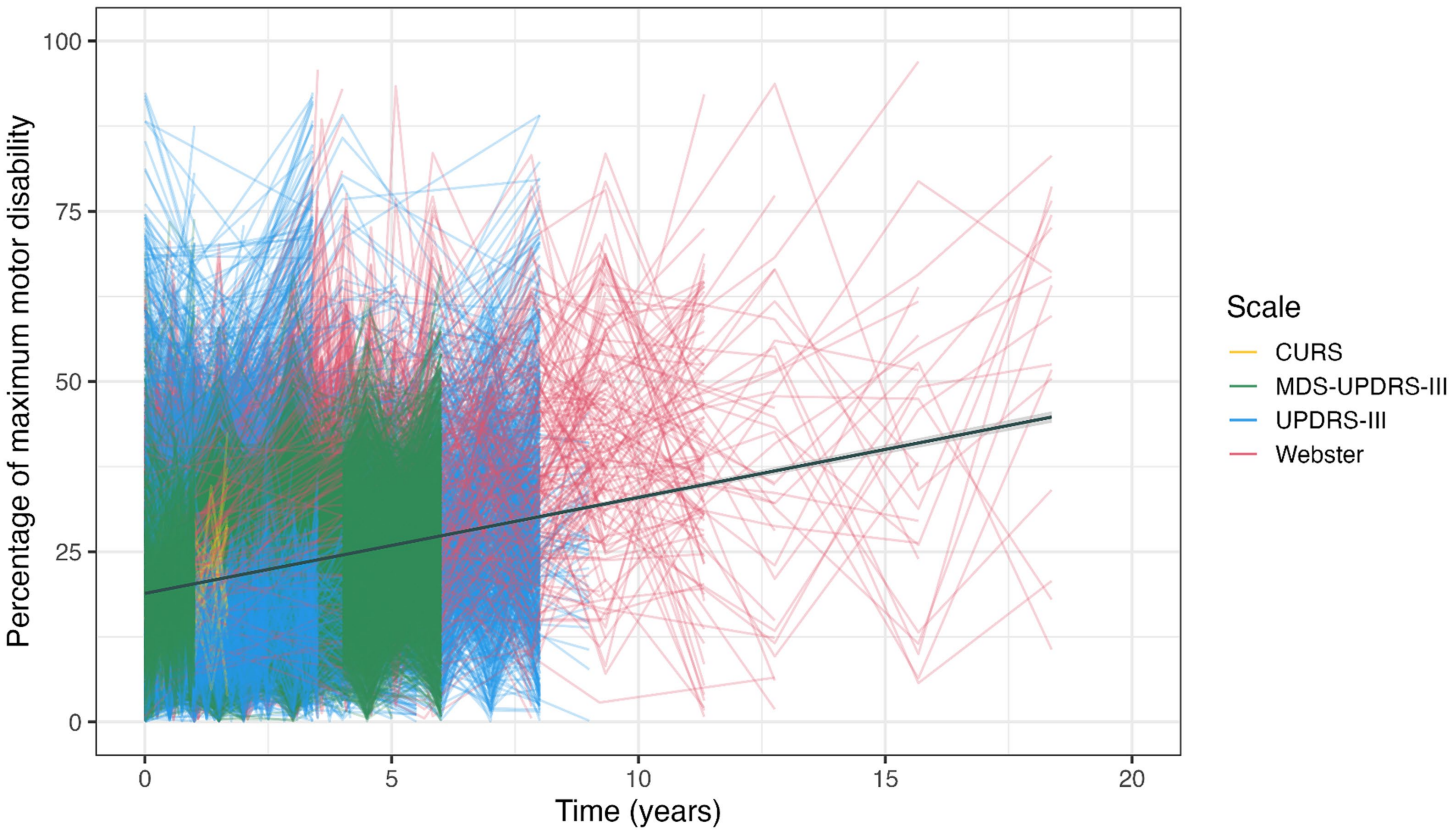
Data simulation. CURS, Columbia University Rating Scale.

Figure 3 presents a data simulation of 61 studies for which we were able to extract sufficient statistical information on individual time point observations. This illustrates the variety of selected studies in terms of relative motor disability and observation period. The use of different objective motor scales and the paucity of observations extending beyond 10 years can both be appreciated. The linear function of best fit has a slope that indicates motor progression of 1.5% p.a. It should be noted that only 4 of the 8 pre-treatment studies were included in the data simulation, which may have contributed to a lower progression rate than in the summary metric of all studies combined.

### 3.3 Determinants of motor progression

Table 1 presents a breakdown by study attribute for all 84 articles with a calculable PD progression rate. The most significant difference concerned untreated motor disability, in 8 articles, which was greater than for all other studies that contained individuals on dopaminergic agents (Comparison 5). The untreated disability rate was based on a combined sample of 1,571 clinical trial participants with a mean baseline PD duration of 1.3 ± 0.7 years, observed for a mean period of 0.9 ± 0.1 years. As all untreated disability articles presented clinical trial results in early PD, they contributed to differences in progression rates shown in Comparisons 2 and 4. Excluding the 8 untreated studies from both analyses narrowed those differences and removed their significance (Comparison 2: median 2.0 ± 1.31% p.a. vs. 1.5 ± 1.4% p.a., *p* = 0.07, *q* = 0.14; Comparison 4: median 2.0 ± 1.9% p.a. vs. 1.6 ± 1.2% p.a., *p* = 0.11, *q* = 0.18).

**Table 1:** Studies with a calculable rate of motor progression, analyzed according to study attributes using the Wilcoxon rank-sum test with correction for multiple comparisons.

	a vs. b	a	b	Wilcoxon rank-sum test
1	Age at baseline <64 years (*n* = 41) vs. ≥64 years (*n* = 39), 4 NA	Median: 1.8 ± 2.5% p.a. Mean: 2.6 ± 2.0% p.a.	Median: 1.6 ± 1.5% p.a. Mean: 1.8 ± 1.7% p.a.	W = 611 *p* = 0.05 *q* = 0.10
2	Baseline disease duration <3 years (*n* = 40) vs. ≥3 years (*n* = 32), 13 NA	Median: 2.2 ± 1.9% p.a. Mean: 2.6 ± 1.9% p.a.	Median: 1.5 ± 1.5% p.a. Mean: 1.8 ± 1.9% p.a.	W = 407 *p* = 0.008 *q* = 0.02
3	Observation period ≤ 3 years (*n* = 47) vs. >3 years (*n* = 37)	Median: 1.8 ± 3.3% p.a. Mean: 2.5 ± 2.2% p.a.	Median: 1.6 ± 0.9% p.a. Mean: 1.8 ± 1.0% p.a.	*p* = 0.26 *q* = 0.39
4	Clinical trial (*n* = 31) vs. cohort study (*n* = 53)	Median: 2.4 ± 3.0% p.a. Mean: 3.0 ± 2.0% p.a.	Median: 1.6 ± 1.2% p.a. Mean: 1.7 ± 1.5% p.a.	W = 1,156 *p* = 0.003 *q* = 0.01
5	Untreated* motor disability (*n* = 8) vs. all other studies (*n* = 76)	Median: 4.5 ± 2.9% p.a. Mean: 4.9 ± 2.1% p.a.	Median: 1.6 ± 1.4% p.a. Mean: 1.9 ± 1.6% p.a.	W = 70 *p* = 0.0004 *q* = 0.003
6	Treatment* commenced ≥25% of sample (*n* = 19) vs. all other studies containing individuals on treatment* (*n* = 58)	Median: 1.5 ± 0.8% p.a. Mean: 1.8 ± 1.0% p.a.	Median: 1.6 ± 1.5% p.a. Mean: 1.9 ± 1.7% p.a.	*p* = 0.93 *q* = 0.93
7	Fixed sample size (*n* = 39) vs. Sample attrition (*n* = 45)	Median: 1.6 ± 1.7% p.a. Mean: 2.0 ± 2.0% p.a.	Median: 1.8 ± 1.2% p.a. Mean: 2.3 ± 1.7% p.a.	*p* = 0.29 *q* = 0.39
8	Prevailing (*n* = 43) vs. defined (*n* = 33) motor state in relation to treatment*	Median: 1.6 ± 1.2% p.a. Mean: 1.7 ± 1.3% p.a.	Median: 1.7 ± 1.4% p.a. Mean: 2.1 ± 1.8% p.a.	*p* = 0.41 *q* = 0.47

*q*-values, *p*-values adjusted for false discovery rate; p.a., per annum. *”Untreated” and “treatment” refer to dopaminergic medication.

Comparison 6 addresses the potentially distorting effect of the commencement of dopaminergic drugs in a substantial number of subjects during an observation period. Comparison 7 looks at ‘real world’ prospective research in which patients are lost to follow-up.

Weighting the progression rate for sample number rendered a mean progression of 1.8% p.a. Weighting for study duration and number of time points gave 1.9% p.a. for each. None of these variables created important bias. Supplementary Figure S2 presents data on progression rate with respect to baseline PD duration.

### 3.4 *On* or *off* scoring

Forty-three studies assessed patients in prevailing motor states (Comparison 8). They showed a small but insignificant reduction in progression rate compared to any study that defined a relationship between motor scoring and the medication cycle. In 24 studies, scoring was performed either *on* or *off* state (see Supplementary Table S2). The average *on* progression rate was 2.1% p.a. (median = 1.6 ± 1.8%), and the average *off* progression was 2.1% p.a. (median = 1.6 ± 1.1%). In a further 9 studies, defined *off* state and levodopa test dose assessments were conducted. Here, *off* exceeded *on* progression in every case but one (average *on* 1.8% p.a., average *off* 2.4% p.a.; median *on* = 1.3 ± 1.2%, median *off* = 2.0 ± 1.2%; paired Wilcoxon rank-sum test *V* = 39, *p* = 0.05, *n* = 18).

### 3.5 Commencement of levodopa treatment

Sixteen studies with concurrent commencement of levodopa met the conditions for calculation of initial response. The mean initial levodopa response was 40.3 ± 15.2% of the pre-treatment disability score. The mean time to maximum improvement in motor score was 7.0 ± 2.3 months in the 10 studies where multiple early time points permitted this to be estimated.

Fifteen articles with concurrent commencement of levodopa had sufficient follow-up to track the progression of motor disability in relation to pre-treatment scores. Pre-treatment disability was exceeded in five studies, after a mean time of 4.5 years. In one of these studies with practically defined *off* state methodology, the intercept of *off* motor score with pre-treatment disability was at 4.9 years. The pre-treatment score had not been reached at the end of the observation period in the other 10 studies (after a mean interval of 3.0 years).

### 3.6 Motor progression of prodromal Parkinson’s disease

Only a handful of studies addressed this topic. Although insufficient for statistical analysis, they provide interesting data on motor function leading up to the time of diagnosable idiopathic PD. One study used olfaction and dopamine transporter scans to define an at-risk sample, back-analyzing motor scores from the time point of phenoconversion (90). The UPDRS motor score worsened at a rate of 7.5% p.a. in the 2 years before diagnosis, whereas progression had been 0.9% p.a. three and more years before diagnosis (90). A cohort identified by REM sleep disorder progressed at 5.2% p.a. over 2 years before phenoconversion (which included individuals who fulfilled diagnostic criteria for dementia with Lewy bodies), compared to 0.6% p.a. three and more years prior to diagnosis (38). Another REM sleep disorder cohort showed a progression rate of 3.2% p.a. for phenoconverters on a 5-year time scale not aligned with the time of PD diagnosis (51). In a study of community-dwelling older adults, subjects with minimal parkinsonian signs by the criteria of Louis et al. (114) progressed only at 0.13% p.a. (63).

### 3.7 Risk of bias assessment

Seventy-seven studies were judged to have a low risk of bias and 13 studies to have a moderate risk (Supplementary Figure S1). Potential sources of bias were related to selection, diagnosis, and outcomes.

### 3.8 Meta-regression and heterogeneity

The annual rate of change data was highly heterogeneous between studies, with I^2^ being 95.6% (CI = 95.0–96.2). Using multivariate linear models, we found no significant influence from motor scale, study duration, study type, sample number, risk of bias assessment, presence of levodopa treatment, relationship to levodopa medication cycle, baseline disease duration, or baseline age on heterogeneity (test of moderator *p*-value >0.05 for all variables).

## 4 Discussion

### 4.1 Motor progression of PD

The long-term rate of motor progression of idiopathic PD is close to 2% p.a. Different methods of statistical handling converged around this number, and factors such as baseline age, sample size, and period of observation had insignificant effects.

Parkinsonian motor disability reflects the underlying loss of neurons in the substantia nigra pars compacta and the resultant dopaminergic deficit. The clinical progression rate from our survey aligns quite well with pathological evidence. In four studies that correlated nigral cell counts with disease duration, the rate of nigral cell loss ranged between 1.2 and 1.9% p.a. with respect to control cell populations (10, 12, 115, 116). While biological processes of growth or decay are often governed by exponential laws, nearly all studies with multiple time points reviewed here showed the linear rate of change and that is how we calculated progression. Linear motor decline accords with most clinicians’ impressions of PD. Exponentiality might be expected to increase the progression rate in longer studies, in studies with greater PD duration at baseline, and in older patients at baseline. In Table 1, the trend is in the opposite direction for each of these subgroup analyses. The main caveat to this conclusion is that few observations on the advanced disease were captured by our publication search.

We tried to examine factors that long-term prospective clinical trials of disease-modifying agents need to contend with. Recruitment in the prodromal or early clinical disease phase anticipates sequential commencement of dopaminergic therapy in a substantial proportion of participants, and eventual attrition of sample size from disability or death. Both might be expected to depress the disease trajectory, through treatment benefit from the first factor and by removal of more rapidly progressing cases by the second. Neither, however, greatly affected the progression rate.

### 4.2 Effect of *on* and *off* scoring

Defined *on* and *off* serial motor assessments were available only from a minority of articles. Prevailing motor state studies showed an insignificant reduction in progression rate compared to defined motor states. Studies that classified motor function as either *on* or *off* showed, perhaps surprisingly, similar progression rates. Some of these measurements were obtained by labeling prevailing motor function on usual treatments as *off* or *on.* Such an approach may result in underscoring of *off* or overscoring of *on*. The more rigorous method used in nine other studies employed practically defined *off* states followed by levodopa test doses with ascertainment of maximum benefit. These articles do suggest that *off* motor progression exceeds *on* progression by a modest degree. The *on* and *off* comparisons also show that the magnitude of the levodopa response does not decline with disease progression, despite increasing disability in both *on* and *off* states.

**Figure 4:**
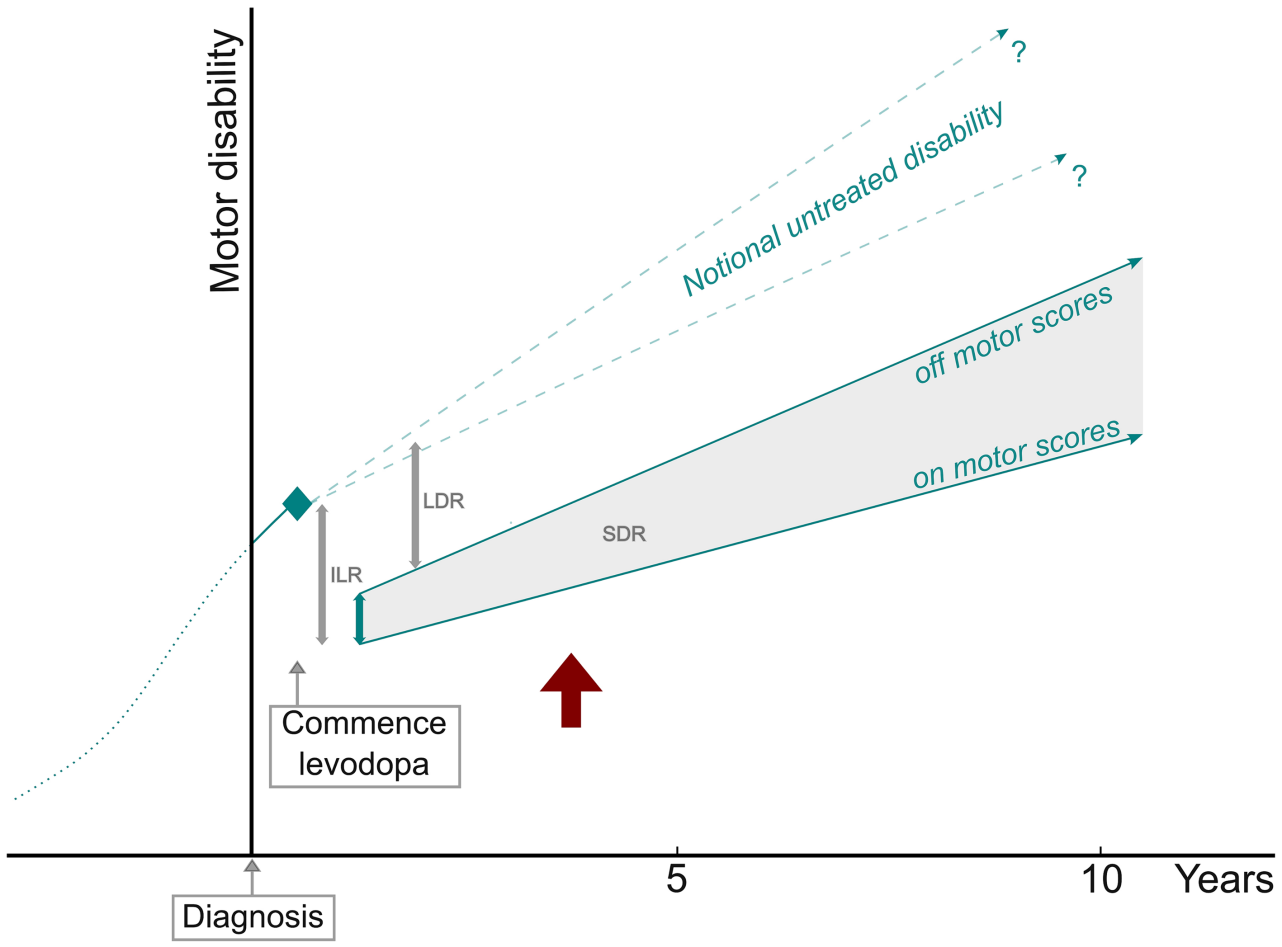
PD motor progression in relation to parameters of the levodopa response. ILR, initial levodopa response; SDR, short-duration response; LDR, long-duration response. *Off* and *on* motor score slopes based on the studies with defined *off* state and levodopa test dose measurements. The red arrow indicates the point at which pre-treatment disability is exceeded.

The motor benefit of levodopa has two components—the short-duration response (SDR), which follows the pharmacokinetic profile of the drug and represents the difference between *on* and *off* scores; and the long-duration response (LDR), which outlasts the elimination of the drug at least by days to weeks, composes 30–50% of the overall response, and is much more difficult to measure (117). While shaped by aggregate data obtained in this review, Figure 4 should not be considered part of the statistical analysis. It is presented with a hypothetical depiction of the LDR as a framework to discuss the motor progression of PD in terms of *on* and *off* measurements of the levodopa response.

### 4.3 Motor progression before dopaminergic treatment and before diagnosis

Although based on a limited number of clinical trials with untreated placebo control participants, there is evidence that motor progression is faster before dopaminergic drugs are commenced. The most statistically significant difference presented in Table 1 is that motor disability increased more than twice as rapidly before treatment. This is unlikely to be an artifact of the lower section of motor scales because, as illustrated in Figure 4, measurements of progression after initial treatment benefit traverse much the same range of scores. While it might be concluded that dopaminergic treatment modifies the motor progression of PD, this does not necessarily mean that some aspect of the underlying pathology is changed. More likely, compensatory processes for nigral cell loss and dopamine deficiency are augmented in some way by exogenous dopamine receptor stimulation.

The idea that certain non-motor features could identify a premonitory phase of PD arose from the Braak model of progression of Lewy pathology and its implication that the substantia nigra is not initially affected (9). With time taken to assemble cohorts with strong risk factors and to follow them long enough to accrue sufficient phenoconversion numbers, there is a relatively small body of research outcomes in this area. Two retrospective back-analyses of phenoconversion in at-risk populations defined in different ways reached a similar conclusion—disability scores in these groups progressed slowly for a time then much more quickly in the 2 years before clinical diagnosis (38, 90). These observations add support to the measurements of untreated PD patients in clinical trials—progression occurs at a rate of more than 4% p.a. in the early motor phase of the disorder (40, 92). In one longitudinal dopamine transporter imaging study, striatal 123-I Ioflupane binding showed greater deterioration in the first year after diagnosis than in subsequent years (118).

### 4.4 Short-and medium-term effects of dopaminergic treatment

After the commencement of levodopa treatment, we calculated that motor scores declined by 40.3% for pre-treatment disability. Lowest scores were recorded at approximately 7 months, after which they begin to track upward with disease progression. A substantial presence of a LDR can be inferred from the fact that progression measurements do not exceed pre-treatment disability for a number of years.

Nutt et al., employing hand tapping as the sole motor assessment, examined the SDR and LDR at the start of dopaminergic treatment. They measured the SDR for the very first exposure to levodopa; this did not greatly change over the next 12 months, during which time the LDR was progressively established (119). Conversely, one study selected for this meta-analysis conducted UPDRS-III scoring for an initial levodopa test dose and then *off* and *on* measurements after 12 months of treatment. The SDR fell to below 50% of the levodopa-naïve magnitude, with a sizable LDR estimated to have developed (32). As shown in Figure 4, a line of notional untreated disability, which would be expected to progress at a certain rate over time, can theoretically help to define the size of the LDR. The gradient of this line is uncertain, though not likely to be less than 2% p.a.

In the medium term, cross-sectional studies of the SDR suggest that its magnitude roughly doubles over 5–10 years of treatment, a trend more pronounced in patients with motor fluctuations (120–122). This predicts that an evolving SDR should delay the onset and reduce the trajectory of the progression of *on* disability, consistent with those articles that scored both *off* and *on* states. A small number of studies identified for this review contained prospective *on* and *off* state measurements extending into the second decade of the disease course (35, 48) and suggested that *on* and *off* scores eventually deteriorate in parallel.

### 4.5 Performance of the motor scales

The older UPDRS-III motor scale (maximum disability score = 108) was the most widely used assessment. The UPDRS-III was discriminatory at scores of less than 10 in prodromal PD and in individuals with minimal parkinsonian signs. In studies that recruited untreated subjects with early PD, there was good consistency at approximately 20% of the maximum disability score (median = 19.3 ± 8.9%). The average UPDRS-III score at phenoconversion to diagnosable PD in two prodromal disease studies was 18.4 (38, 90). Five motor scales were included in our review. There were no significant differences in progression rate according to scale type.

### 4.6 Study limitations

Our broad inclusion criteria selected some studies that were small, were relatively short, had been conducted many years ago, or presented data with insufficient statistical detail for meta-analysis. Nevertheless, we found a consistent pattern of PD progression across a wide range of study types.

The finding that the rate of PD progression appears to be twice as fast in untreated compared to treated patients is both interesting and unexpected. These findings were drawn from methodologically different studies and diverse cohorts. The question is worthy of future examination by double-blind trials designed specifically to address it.

We found a high degree of heterogeneity. Between-study variability can be caused by clinical or methodological diversity (123). With published diagnostic criteria for PD employed by most included articles, clinical heterogeneity from patient selection seems unlikely. We performed mixed model analyses with several moderator variables, none of which could explain a large amount of heterogeneity. Presumably, non-quantifiable between-study methodological diversities were responsible. Although we found no statistically significant influence of motor scale on heterogeneity, it is still possible that combining scoring systems with different scaling properties had some effect. For instance, the UPDRS-III and MDS-UPDRS-III differ from some older scales in emphasis given to bradykinesia over other motor signs, and in their balance of “lateralized” versus “midline” motor items.

Many articles had to be screened for this review, and it is possible that some eligible studies were overlooked. Our study quality assessment, which identified no studies with a high risk of bias, employed a version of the CASP cohort checklist. This had to be applied to studies that did not have our research question as a primary objective.

### 4.7 Practical implications for long-term measurement of motor progression

1. PD progresses at roughly 2% p.a. in the long term. A neuroprotective treatment that slows disease progression by, say, 30% would be hard to detect over a short observation period.
2. While study designs involving prodromal or untreated PD avoid some confounding effects of symptomatic treatment, different assumptions about the rate of disease progression may be needed. Motor progression appears to be faster before than after dopaminergic drugs are commenced.
3. The effects of starting dopaminergic treatment are substantial and complex. They include the establishment of the LDR, though its presence and magnitude can only be inferred in most circumstances.
4. Practically defined *off* state measurements probably give the best guide to progression once dopaminergic therapy has begun. A defined *off* state with levodopa test dose method maximizes information about the medication cycle.
5. It is possible to conduct accurate long-term observations of motor progression that span prodromal, early, and treated disease phases and allow for serial commencement of dopaminergic drugs and sample attrition over time.
6. While simultaneously registering symptomatic treatment effects and underlying disease progression, the objective motor scales are sensitive to small changes in disability. Motor scores show linear temporal relationships throughout the early and middle stages of PD. Significant floor effects in these assessment tools were not apparent in our analysis.

## Data Availability

The original contributions presented in the study are included in the article/Supplementary material, further inquiries can be directed to the corresponding author.
