# Supplementary material for: Rate of motor progression in Parkinson’s disease: a systematic review and meta-analysis
Source: Front Neurol. 2024 Sep 26;15:1452741. doi: 10.3389/fneur.2024.1452741 (PMC11464440; doi:10.3389/fneur.2024.1452741)
Supplement: Supplementary file 1 [file Data_Sheet_1.docx]

**APPENDIX 1**

**Database Ovid MEDLINE**

*Search strategy 1:* to capture all studies on motor progression in Parkinson’s disease.

Ovid MEDLINE(R) ALL <1946 to March 15, 2023>

1 Parkinson Disease/ 80482

2 Parkinson.mp. 99452

3 Parkinson's disease.mp. 104428

4 Disease progression/ 188450

5 Progression.mp. 763537

6 Decline.mp. 251316

7 Disability.mp. 274095

8 Disability evaluation/ 51252

9 Observational study/ 139325

10 Cohort studies/ 325925

11 Randomi?ed controlled trial.mp. 642189

12 Follow-up.mp. 1555639

13 Longitudinal.mp. 379502

14 Prospective.mp. 950444

15 Natural history.mp. 55045

16 1 or 2 or 3 140089

17 4 or 5 or 6 or 7 or 8 1253417

18 9 or 10 or 11 or 12 or 13 or 14 or 15 3238712

19 16 and 17 and 18 3698

20 limit 19 to (english language and humans) 3155

21 Review.pt. 3122161

22 20 not 21 **2892**

*Search strategy 2:* to capture all studies on the relationship of motor progression with pharmacological treatment, and the evolution of the levodopa response.

Ovid MEDLINE(R) ALL <1946 to March 15, 2023>

1 Levodopa/ 17586

2 Levodopa.mp. 23549

3 Parkinson Disease/ 80836

4 Parkinson.mp. 99833

5 Parkinson's disease.mp. 105050

6 Disease progression/ 188775

7 Progression.mp. 768047

8 Decline.mp. 252728

9 Disability.mp. 275370

10 Motor response.mp. 4459

11 Long-duration response.mp. 59

12 Short-duration response.mp. 56

13 Dyskinesia.mp. 19218

14 1 or 2 23549

15 3 or 4 or 5 140773

16 6 or 7 or 8 or 9 or 10 or 11 or 12 or 13 1282451

17 14 and 15 and 16 5497

18 limit 17 to (english language and humans) 3762

19 Review.pt. 3135552

20 18 not 19 **2700**

**Database Ovid EMBASE**

Embase <1974 to 2023 March 15>

1 Parkinson disease/ 188218

2 Parkinson's disease.mp. 158381

3 Parkinson.mp. 204871

4 Progression.mp. 1097950

5 Decline.mp. 347021

6 Disability.mp. 379460

7 Longitudinal.mp. 487756

8 Cohort.mp. 1557767

9 Natural history.mp. 78089

10 observational study/ 319904

11 randomized controlled trial/ 775463

12 1 or 2 or 3 228349

13 4 or 5 or 6 1768766

14 Review.pt. 3067408

15 7 or 8 or 9 or 10 or 11 2939265

16 12 and 13 and 15 5732

17 limit 16 to (human and english language) 5457

18 17 not 14 **5183**

**Database Cochrane Central Register of Clinical Trials**

Searched on 2023 March 15.

1 Parkinson* 14694

2 Progression OR decline OR disability* 154502

3 Observational study OR cohort* OR randomi?ed controlled trial OR follow-up OR longitudinal OR prospective OR natural history 1363212

4 #1 AND #2 AND #3 1936

5 Excluding reviews **1551**

**Database clinicaltrials.gov**

Searched on 2023 March 15. Filtered on “Parkinson’s disease” and “results available” resulted in **432** records.
