# Supplementary material for: Rate of motor progression in Parkinson’s disease: a systematic review and meta-analysis
Source: Front Neurol. 2024 Sep 26;15:1452741. doi: 10.3389/fneur.2024.1452741 (PMC11464440; doi:10.3389/fneur.2024.1452741)
Supplement: Supplementary file 2 [file Table_1.DOCX]

**Supplementary Table 1:** Adapted version of Critical Appraisal Skills Programme (CASP)^21^ cohort study checklist, which was also applied to placebo arms of clinical trials.

| **Adapted CASP tool** | |
| --- | --- |
| 1. Clearly focused study population? | a. Adults with PD |
| 2. No selection bias? | a. Represents the population of interest  b. Origin of study group stated clearly c. Information on PD duration available |
| 3. Ascertainment of diagnosis? | a. Validated diagnostic criteria for Parkinson's disease specified |
| 4. Adequate measurement of outcomes? | a. Objective outcome measure defined and referenced b. Relationship of motor assessment to medication cycle stated c. Low risk of attrition bias: dropout rate not excessive, reasons for dropout provided d. Observation period >3 years e. Three or more time points, including baseline f. Patient number >50 |
| 5. Results | a. Mean and standard deviation (or values from which these can be calculated) available on each timepoint in published material |
| DETERMINATION OF RISK OF BIAS | Based on criteria listed above, each question was allocated a grading for risk of bias: low (green), moderate (orange) and high (red).  The overall risk of bias for an article was determined according to the following formulae:  Low risk of bias:   - 1 yellow, 4 green - 2 yellow, 3 green - 1 red, 1 yellow, 3 green   Moderate risk of bias:   - 3 yellow, 2 green - 4 yellow, 1 green - 1 red, 2 yellow, 2 green - 2 red 2 yellow, 1 green   High risk of bias:   - 3 or more red |

**Supplementary Table 2.** Study characteristics. R = calculable rate of motor progression, M = meta-analysis, S = data simulation, C = simultaneous commencement of levodopa, P = prodromal; PD = Parkinson’s disease; p.a. = per annum; n/a = not available.

| **Author, year** | **Analysis** | **Study type** | **Baseline sample size** | **Baseline age (years)** | **Baseline PD duration (years)** | **Study duration (years)** | **Motor scale (maximum score)** | **Motor assessment in relation to medication cycle** | **Motor progression rate** |
| --- | --- | --- | --- | --- | --- | --- | --- | --- | --- |
| Alarcon, 1998 ^24^ | R, C | Clinical trial | 38 | 64 | 4.1 | 3.0 | UPDRS-III (108) | Prevailing | 0.4% p.a. |
| Allain, 2000 ^25^ | R, M, S | Clinical trial | 41 | 60 | 1.7 | 5.0 | UPDRS-III (108) | Prevailing | 1.0% p.a. |
| Alves, 2005 ^26^ | R, M, S | Population | 232 | 74 | 9.1 | 8.0 | UPDRS-III (108) | Prevailing | 2.2% p.a. |
| Antonini, 2012 ^27^ | R, M, S | Cohort | 707 | 67 | n/a | 2.0 | UPDRS-III (108) | Prevailing | 1.6% p.a. |
| Athauda, 2017 ^28^ | R, M, S | Clinical trial | 29 | 58 | 6.4 | 1.2 | MDS-UPDRS-III (132) | Off | 1.3% p.a. |
|  |  |  |  |  |  |  |  | On | 0.2% p.a. |
| Athauda, 2022 ^29^ | R, M, S | Cohort | 1930 | 68 | 1.3 | 3.0 | MDS-UPDRS-III (132) | Prevailing | 1.7% p.a. |
| Aviles-Olmos, 2013 ^101^ | R, M, S | Clinical trial | 24 | 59 | 11 | 1 | MDS-UPDRS-III (132) | Off | 1.7% p.a. |
|  |  |  |  |  |  |  |  | On | 2.7% p.a. |
| Ayala, 2017 ^30^ | R, M, S | Cohort | 205 | 66 | 8.1 | 3.0 | SCOPA mot 42 (30) | Prevailing | 1.6% p.a. |
| Carvalho, 2023 ^31^ | R, M, S | Cohort | 98 | 68 | 4.6 | 2.0 | MDS-UPDRS-III (132) | On | 0.9% p.a. |
| Chan, 2023 ^105^ | R, M, S | Cohort | 103 | 68 | 2.7 | 1 | UPDRS-III (108) | Prevailing | -1.2% p.a. |
| Cilia, 2020 ^32^ | R, M, S, C | Cohort | 30 | 64 | 7.1 | 2.0 | UPDRS-III (108) | Off | 3.4% p.a. |
|  |  |  |  |  |  |  |  | On | 1.3% p.a. |
| Davidson, 2012 ^33^ | C | Population | 133 | 71 | n/a | 1.0 | UPDRS-III (108) | n/a | n/a |

**Supplementary Table 2** (continued).

| **Author, year** | **Analysis** | **Study type** | **Baseline sample size** | **Baseline age (years)** | **Baseline PD duration (years)** | **Study duration (years)** | **Motor scale (maximum score)** | **Motor assessment in relation to medication cycle** | **Motor progression rate** |
| --- | --- | --- | --- | --- | --- | --- | --- | --- | --- |
| Davis, 2016 ^102^ | R, M | Cohort | 733 | 68 | 8.7 | 3.0 | MDS-UPDRS-III (132) | Prevailing | 1.6% p.a. |
| Deng, 2019 ^34^ | R, M, S | Cohort | 156 | 65 | 0.2 | 9.0 | UPDRS-III (108) | On | 1.0% p.a. |
| Devos, 2022 ^108^ | R, M, S | Clinical trial | 186 | 63 | 1.1 | 0.7 | MDS-UPDRS-III (132) | Untreated | 4.4% p.a. |
| Ding, 2016 ^35^ | R, M, S, C | Cohort | 34 | 64 | 1.7 | 20.3 | Mod Webster (36) | Off | 2.0% p.a. |
|  |  |  |  |  |  |  |  | On | 1.3% p.a. |
| Duarte Folle, 2019 ^41^ | R, M, S | Cohort | 776 | 71 | 3.0 | 3.4 | UPDRS-III (108) | Off | 0.0% p.a. |
| Dupont, 1996 ^36^ | R, M, S | Clinical trial | 69 | 66 | 2.7 | 5.0 | Webster (30) | On | 5.0% p.a. |
| Eggers, 2012 ^37^ | R, M, S | Cohort | 27 | 59 | 3.8 | 2.5 | UPDRS-III (108) | Off | 1.8% p.a. |
|  |  |  |  |  |  |  |  | On | 1.2% p.a. |
| Evans, 2011 ^38^ | R | Population | 122 | n/a | 0.4 | 7.9 | UPDRS-III (108) | On | 2.1% p.a. |
| Fahn (PSG), 2004 ^39^ | R, M | Clinical trial | 90 | 65 | 0.4 | 0.8 | UPDRS-III (108) | Untreated | 6.9% p.a. |
| Fereshtehnejad, 2019 ^40^ | P | Cohort | 154 | n/a | n/a | n/a | UPDRS-III (108) | n/a | n/a |
| Frazzitta, 2012 ^42^ | R, M, S | Clinical trial | 25 | 70 | 9.0 | 1.0 | UPDRS-III (108) | On | 5.6% p.a. |
| Fu, 2022 ^43^ | R, M, S | Cohort | 234 | 66 | 5.0 | 5.0 | UPDRS-III (108) | On | 0.4% p.a. |
| Gago, 2009 ^44^ | R, M | Cohort | 24 | 64 | 5.0 | 6.0 | UPDRS-III (108) | Prevailing | 3.8% p.a. |
| García, 2022 ^88^ | R, M | Cohort | 511 | 63 | 5.3 | 2.0 | UPDRS-III (108) | Off | 1.5% p.a. |
| García-Ruiz, 2004 ^45^ | R, M, S | Cohort | 59 | 62 | n/a | 5.0 | UPDRS-III (108) | Prevailing | 1.3% p.a. |

**Supplementary Table 2** (continued). *Patients were assessed in both *off* and *on* state, but this was not defined *off* and *on* after testdose.

| **Author, year** | **Analysis** | **Study type** | **Baseline sample size** | **Baseline age (years)** | **Baseline PD duration (years)** | **Study duration (years)** | **Motor scale (maximum score)** | **Motor assessment in relation to medication cycle** | **Motor progression rate** |
| --- | --- | --- | --- | --- | --- | --- | --- | --- | --- |
| Helmy, 2022 ^46^ | R, M, S | Cohort | 45 | 57 | 4.9 | 1.0 | MDS-UPDRS-III (132) | Off | 8.8% p.a. |
| Hely, 1994 ^47^ | R, C | Clinical trial | 64 | 62 | 2.1 | 5.0 | Mod Columbia (102) | Prevailing | 1.6% p.a. |
| Holden, 2018 ^48^ | R, M, S | Cohort | 362 | 61 | n/a | 5.0 | MDS-UPDRS-III (132) | Off | 1.8% p.a. |
| Holloway, 2004 ^49^ | R, M, S, C | Clinical trial | 150 | 61 | 1.8 | 4.0 | UPDRS-III (108) | Prevailing | 1.7% p.a. |
| Hughes, 1994 ^50^ | R | Cohort | 23 | 57 | 12.0 | 3.1 | Mod Webster (36) | Off | 4.5% p.a. |
|  |  |  |  |  |  |  |  | On | 4.3% p.a. |
| Imarisio, 2022 ^51^ | R, M, S | Cohort | 71 | 65 | 5.2 | 2.0 | MDS-UPDRS-III (132) | Prevailing | 0.6% p.a. |
| Jankovic, 2001 ^52^ | R | Cohort | 297 | 61.6 | 6.5 | 6.4 | UPDRS-III (108) | Off *  On * | 1.3% p.a.  0.7% p.a. |
| Joza, 2023 ^53^ | P | Cohort | 1160 | n/a | n/a | 3.3 | MDS-UPDRS-III (132) | n/a | n/a |
| Katzenschlager, 2008 ^54^ | R, M, S, C | Clinical trial | 249 | 57 | 1.6 | 12.0 | Mod Webster (36) | Prevailing | 2.4% p.a. |
| Kraus, 2005 ^55^ | R, M, S | Cohort | 411 | 62 | n/a | 4.0 | Webster (30) | Prevailing | 2.0% p.a. |
| Lang, 2022 ^109^ | R, M, S | Clinical trial | 100 | 61 | 0.7 | 1.0 | MDS-UPDRS-III (132) | Untreated | 4.6% p.a. |
| Larsen, 1999 ^56^ | R, M, S, C | Clinical trial | 81 | 64 | 2.0 | 5.0 | UPDRS-III (108) | On | 2.0% p.a. |
| Lenfeldt, 2013 ^57^ | R, M, S | Cohort | 66 | 69 | n/a | 5.0 | UPDRS-III (108) | Prevailing | 0.8% p.a. |
| Lewis, 2020 ^58^ | R, M | Cohort | 80 | 68 | 3.8 | 3.0 | MDS-UPDRS-III (132) | On | 0.0% p.a. |
| Li, 2018 ^59^ | R, M, S | Cohort | 23 | 55 | 5.6 | 1.6 | UPDRS-III (108) | Off | 2.4% p.a. |
| Lopez, 2010 ^60^ | R, M, S | Cohort | 64 | n/a | n/a | 10.0 | UPDRS-III (108) | Prevailing | 1.5% p.a. |
| Louis, 1999 ^61^ | R | Cohort | 237 | 73 | 6.8 | 7.0 | modUPDRS-III (100) | Prevailing | 1.5% p.a. |

**Supplementary Table 2** (continued).

| **Author, year** | **Analysis** | **Study type** | **Baseline sample size** | **Baseline age (years)** | **Baseline PD duration (years)** | **Study duration (years)** | **Motor scale (maximum score)** | **Motor assessment in relation to medication cycle** | **Motor progression rate** |
| --- | --- | --- | --- | --- | --- | --- | --- | --- | --- |
| Lubomski, 2022 ^62^ | R, M, S | Cohort | 82 | 67 | 8.6 | 1.0 | MDS-UPDRS-III (132) | On | 4.5% p.a. |
| Ludin, 1976 ^63^ | R, M, S, C | Cohort | 35 | 56 | n/a | 3.0 | Webster (30) | Prevailing | 4.5% p.a. |
| Maple-Grødem, 2021 ^104^ | R, M | Population | 440 | 70 | 2.1 | 7 | UPDRS-III (108) | Prevailing | 1.3% p.a. |
| Melzer, 2015 ^110^ | R, M | Cohort | 23 | 70 | 5.6 | 1 | MDS-UPDRS-III (132) | Prevailing | -1.4% p.a. |
| Merola, 2016 ^64^ | R, M, S | Cohort | 20 | 62 | 14.1 | 5.1 | UPDRS-III (108) | Off | 2.5% p.a. |
|  |  |  |  |  |  |  |  | On | 0.9% p.a. |
| Miller-Patterson, 2020 ^65^ | P | Population | 205 | 83 | n/a | 3.8 | UPDRS-III (108) | n/a | n/a |
| Moccia, 2016 ^66^ | R, M, S | Cohort | 79 | 64 | 1.2 | 4 | UPDRS-III (108) | Off | 1.7% p.a. |
| Mollenhauer, 2019 ^67^ | R, M, S | Cohort | 135 | 65 | n/a | 4.0 | MDS-UPDRS-III (132) | Prevailing | 1.4% p.a. |
| Muslimović, 2009 ^14^ | R, M, S | Cohort  (new onset) | 116 | 65 | 1.5 | 3.0 | UPDRS-III (108) | On | 2.5% p.a. |
|  |  | Cohort (established) | 64 | 65 | 6.4 | 3.0 | UPDRS-III (108) | On | 0.6% p.a. |
| Myllylä, 1992 ^68^ | R, M, S | Clinical trial | 25 | 61 | 1.8 | 1.0 | CURS (100) | Untreated | 6.7% p.a. |
| Myllylä, 1995 ^69^ | R, M, S, C | Clinical trial | 21 | 60 | 1.7 | 2.0 | CURS (100) | Prevailing | 3.2% p.a. |
| Ng, 2015 ^70^ | R, M, S | Cohort | 81 | 65 | 5.4 | 1.5 | UPDRS-III (108) | Prevailing | 0.9% p.a. |
| O'Suilleabhain, 2006 ^71^ | R, M, S | Cohort | 79 | 65 | 3.7 | 2.0 | UPDRS-III (108) | Prevailing | 0.3% p.a. |
| Oertel, 2006 ^72^ | R, M, S, C | Clinical trial | 146 | 59 | n/a | 3.0 | UPDRS-III (108) | Prevailing | 1.3% p.a. |
| Olanow, 1995 ^73^ | R, M, S, C | Clinical trial | 21 | 66 | 3.0 | 1.0 | UPDRS-III (108) | Prevailing | 0.5% p.a. |

**Supplementary Table 2** (continued).

| **Author, year** | **Analysis** | **Study type** | **Baseline sample size** | **Baseline age (years)** | **Baseline PD duration (years)** | **Study duration (years)** | **Motor scale (maximum score)** | **Motor assessment in relation to medication cycle** | **Motor progression rate** |
| --- | --- | --- | --- | --- | --- | --- | --- | --- | --- |
| Olanow, 2006 ^74^ | R | Clinical trial | 71 | 61 | 0.9 | 1.0 | UPDRS-III (108) | Prevailing | 4.8% p.a. |
| Ou, 2021 ^75^ | R, M, S | Cohort | 224 | 58 | 1.5 | 3.0 | UPDRS-III (108) | Prevailing | 1.8% p.a. |
| Pagano, 2022 ^107^ | R, M, S | Clinical trial | 105 | 60 | 0.8 | 1 | MDS-UPDRS-III (132) | Untreated | 4.2% p.a. |
| Palermo, 2021 ^76^ | R, M, S | Cohort | 104 | n/a | n/a | 4.0 | MDS-UPDRS-III (132) | Off | 1.2% p.a. |
| Pålhagen, 1998 ^78^ | R, M | Clinical trial | 76 | 64 | 1.9 | 1.0 | UPDRS-III (108) | Untreated | 2.4% p.a. |
| Pålhagen, 2007 ^77^ | R, M, S, C | Clinical trial | 68 | 64 | 1.9 | 6.5 | UPDRS-III (108) | On | 2.4% p.a. |
| Pilotto, 2021 ^100^ | R, M, S | Cohort | 92 | 66 | 5.5 | 2.0 | MDS-UPDRS-III (132) | Prevailing | 0.9% p.a. |
| Pirker, 2003 ^79^ | R, M, S | Cohort | 21 | 56 | 2.4 | 5.3 | UPDRS-III (108) | Off | 2.8% p.a. |
|  |  |  |  |  |  |  |  | On | 2.4% p.a. |
| Rascol, 2000 ^81^ | R, M, S, C | Clinical trial | 89 | 63 | 2.4 | 5.0 | UPDRS-III (108) | Prevailing | 0.8% p.a. |
| Rascol, 2011 ^5^ | R | Clinical trial | 588 | n/a | n/a | 0.7 | UPDRS-III (108) | Untreated | 1.9% p.a. |
| Ravina, 2012 ^82^ | R, M, S | Cohort | 491 | 60 | 0.8 | 5.5 | UPDRS-III (108) | Prevailing | 0.8% p.a. |
| Reinoso, 2014 ^83^ | R, M, S | Cohort | 576 | 64 | n/a | 9.0 | UPDRS-III (108) | On | 1.2% p.a. |
| Rinne, 1997 ^84^ | C | Clinical trial | 205 | 63 | 2.0 | 1.0 | UPDRS-III (108) | n/a | n/a |
| Rinne, 1998 ^85^ | R, C | Clinical trial | 204 | 61 | 1.9 | 4.0 | UPDRS-III (108) | Prevailing | 2.0% p.a. |
| Ritz, 2012 ^106^ | R, M | Population | 222 | 69 | 1.9 | 5.1 | UPDRS-III (108) | Off | 2.4% p.a. |
| Ryu, 2022 ^86^ | R, M, S | Cohort | 120 | 65 | 0.7 | 3.1 | UPDRS-III (108) | Prevailing | 1.2% p.a. |

**Supplementary Table 2** (continued).

| **Author, year** | **Analysis** | **Study type** | **Baseline sample size** | **Baseline age (years)** | **Baseline PD duration (years)** | **Study duration (years)** | **Motor scale (maximum score)** | **Motor assessment in relation to medication cycle** | **Motor progression rate** |
| --- | --- | --- | --- | --- | --- | --- | --- | --- | --- |
| Santaella, 2020 ^87^ | R, M | Cohort | 46 | 58 | 3.0 | 3.0 | UPDRS-III (108) | Off | 0.5% p.a. |
| Schenkman, 2018 ^89^ | R, M, S | Clinical trial | 40 | 64 | 0.4 | 0.5 | MDS-UPDRS-III (132) | Prevailing | 4.8% p.a. |
| Schrag, 2007 ^90^ | R, M, S | Cohort | 145 | 67 | 9.3 | 1.0 | UPDRS-III (108) | Prevailing | 2.7% p.a. |
| Schreiner, 2019 ^91^ | R, M, S | Cohort | 129 | 63 | 5.4 | 4.6 | UPDRS-III (108) | Prevailing | 1.0% p.a. |
| Shoulson, 2007 ^103^ | R, M | Clinical trial | 191 | 60 | 0.9 | 1.8 | UPDRS-III (108) | Prevailing | 2.9% p.a. |
| Shoulson (PSG), 1989 ^4^ | R, M, | Clinical trial | 401 | 61 | 2.2 | 1.0 | UPDRS-III (108) | Untreated | 7.9% p.a. |
| Siderowf, 2020 ^92^ | P | Cohort | 303 | n/a | n/a | 6.3 | UPDRS-III (108) | n/a | n/a |
| Simuni (NINDS), 2015 ^93^ | R, M | Clinical trial | 71 | 59 | 2.3 | 0.8 | UPDRS-III (108) | Prevailing | 4.2% p.a. |
| Simuni (PSG), 2020 ^80^ | R, M, S | Clinical trial | 166 | 62 | 0.9 | 3.0 | UPDRS-III (108) | Off | 1.4% p.a. |
| Sleeman, 2017 ^94^ | R, M, S | Cohort | 145 | 66 | 0.5 | 3.0 | MDS-UPDRS-III (132) | Prevailing | 2.9% p.a. |
| Suzuki, 2013 ^95^ | R, M | Clinical trial | 58 | 71 | 1.1 | 1.0 | UPDRS-III (108) | Prevailing | 1.0% p.a. |
| Velseboer, 2013 ^96^ | R, M, S | Cohort | 129 | 67 | 1.7 | 5.0 | UPDRS-III (108) | Prevailing | 2.0% p.a. |
| Vogt, 2011 ^97^ | R, M, S | Cohort | 44 | 59 | 2.0 | 3.7 | UPDRS-III (108) | Off | 2.6% p.a. |
| Yu, 2020 ^98^ | R, M, S | Cohort | 246 | 57 | 4.8 | 2.0 | MDS-UPDRS-III (132) | Prevailing | 1.7% p.a. |
| Zangaglia, 2009 ^99^ | R, M, S | Clinical trial | 33 | 63 | 10.0 | 3.0 | UPDRS-III (108) | Off | 1.7% p.a. |
|  |  |  |  |  |  |  |  | On | 1.4% p.a. |
