# Supplementary material for: Rate of motor progression in Parkinson’s disease: a systematic review and meta-analysis
Source: Front Neurol. 2024 Sep 26;15:1452741. doi: 10.3389/fneur.2024.1452741 (PMC11464440; doi:10.3389/fneur.2024.1452741)
Supplement: Supplementary file 3 [file Table_2.DOCX]

**Suppl. figure 1:** Risk of bias assessment of included studies, using adapted version of Critical Appraisal Skills Programme (CASP) cohort study checklist and display conventions of Arrias et al.^a^ 1. Study population; 2. Selection bias; 3. Ascertainment of diagnosis; 4. Outcomes; 5. Results.

**
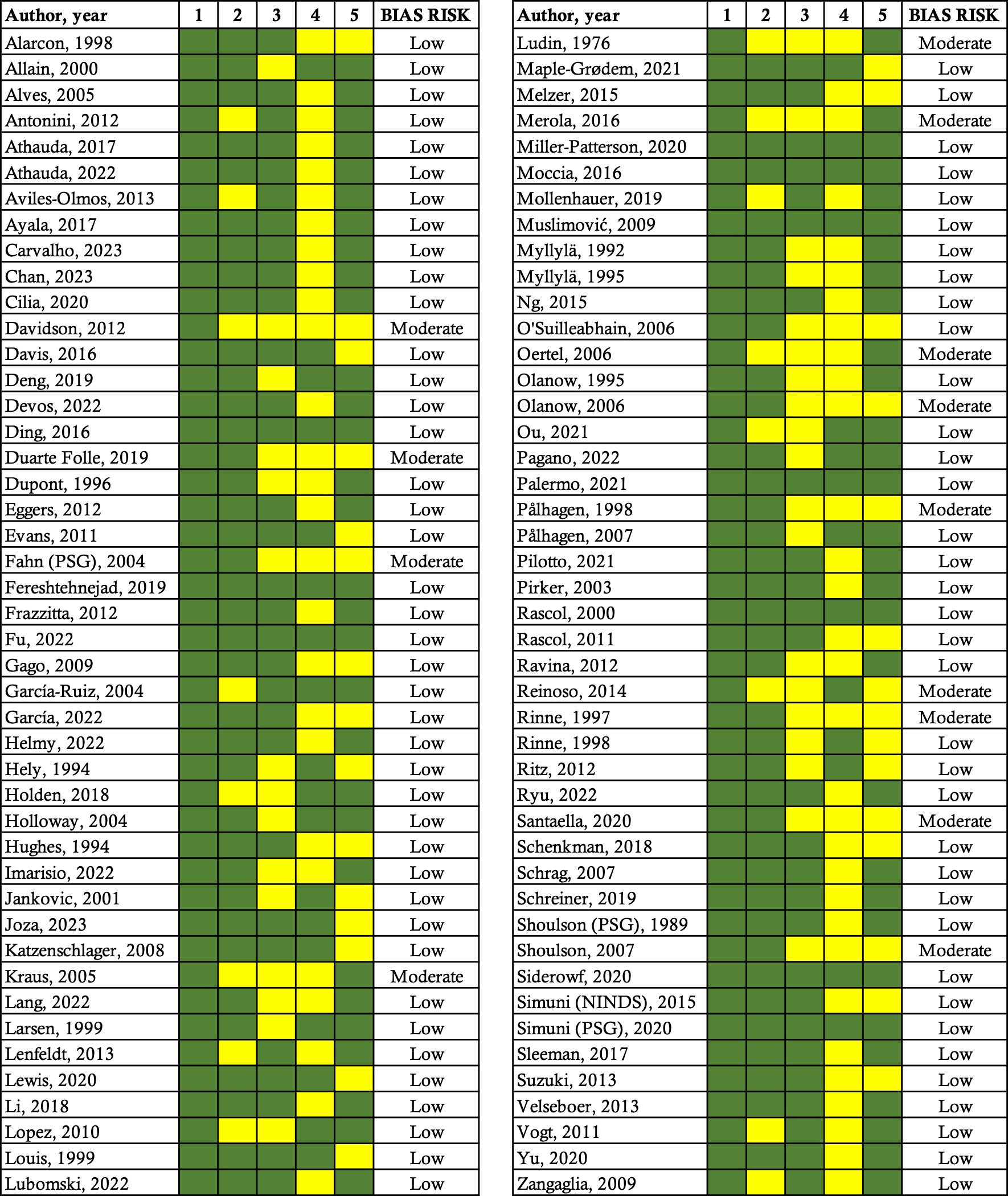
**

**Suppl. figure 2:** Motor progression rate (% p.a.) stratified by baseline PD disease duration (< 5 years, 5-10 years, > 10 years). n/a = not available.

**
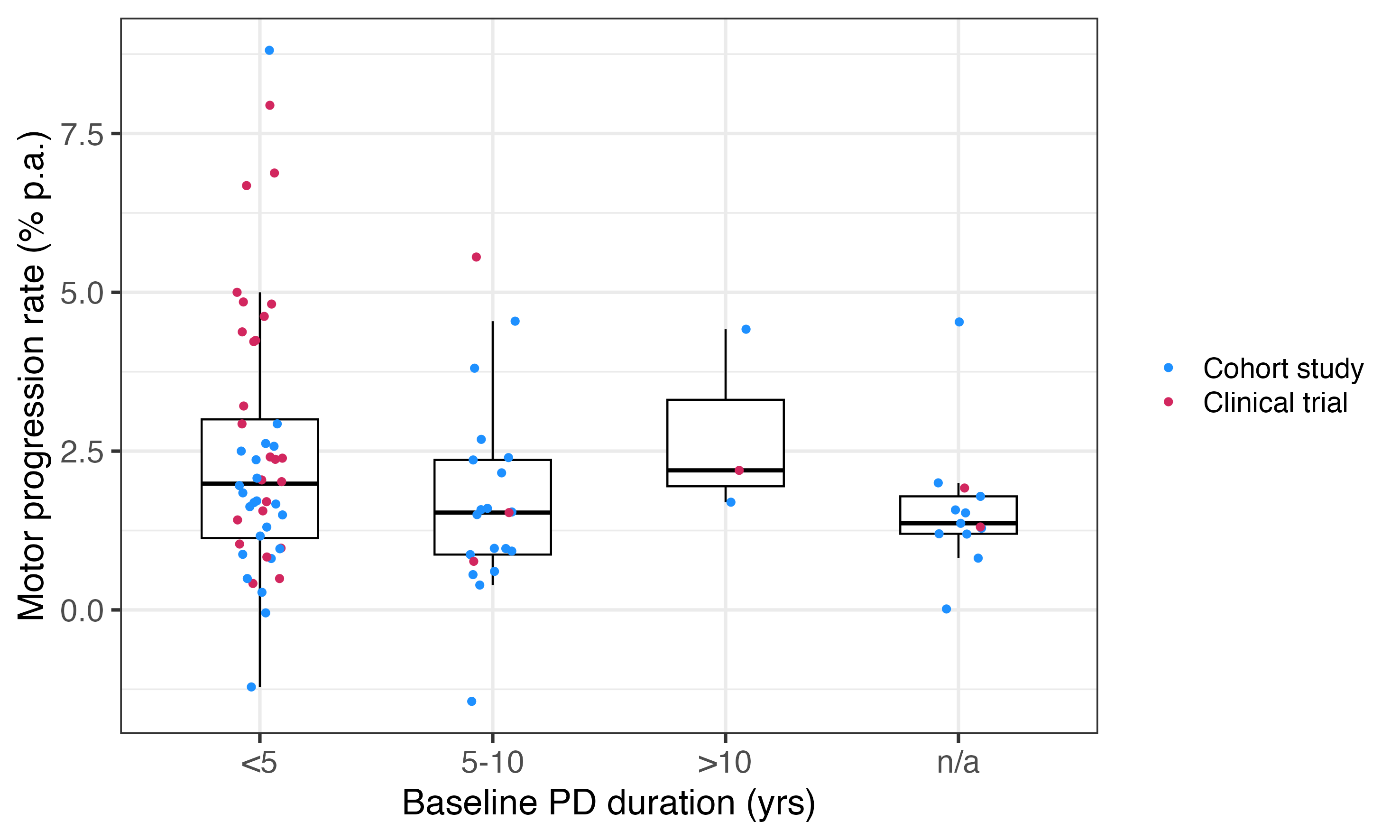
Reference:**

a. Arrais M, Maricoto T, Nwaru BI, et al. Helminth infections and allergic diseases: systematic review and meta-analysis of the global literature. J Allergy Clin Immunol 2022; 149: 2139–2152.
